# Posing for a picture: vesicle immobilization in agarose gel

**DOI:** 10.1038/srep25254

**Published:** 2016-05-03

**Authors:** Rafael B. Lira, Jan Steinkühler, Roland L. Knorr, Rumiana Dimova, Karin A. Riske

**Affiliations:** 1 Departamento de Biofísica, Universidade Federal de São Paulo, São Paulo, Brazil; 2 Department of Theory and Bio-Systems, Max Planck Institute of Colloids and Interfaces, Potsdam, Germany

## Abstract

Taking a photo typically requires the object of interest to stand still. In science, imaging is potentiated by optical and electron microscopy. However, living and soft matter are not still. Thus, biological preparations for microscopy usually include a fixation step. Similarly, immobilization strategies are required for or substantially facilitate imaging of cells or lipid vesicles, and even more so for acquiring high-quality data via fluorescence-based techniques. Here, we describe a simple yet efficient method to immobilize objects such as lipid vesicles with sizes between 0.1 and 100 μm using agarose gel. We show that while large and giant unilamellar vesicles (LUVs and GUVs) can be caged in the pockets of the gel meshwork, small molecules, proteins and micelles remain free to diffuse through the gel and interact with membranes as in agarose-free solutions, and complex biochemical reactions involving several proteins can proceed in the gel. At the same time, immobilization in agarose has no adverse effect on the GUV size and stability. By applying techniques such as FRAP and FCS, we show that the lateral diffusion of lipids is not affected by the gel. Finally, our immobilization strategy allows capturing high-resolution 3D images of GUVs.

Microscopy imaging of cellular and model membranes has revealed a wealth of information about membrane structure and properties. As such, examples include measurements of diffusion coefficient of lipids^1^ and membrane proteins^2^, imaging of membrane domains^3^, and extraction of mechanical information^4^ *in vitro* and *in vivo*. Due to the complex nature and dynamics of living cells, these parameters are commonly studied on model membrane systems, in which the properties are easily controlled by the appropriate choice of their components. Such models include lipid monolayers, supported and black lipid bilayers, lipid-based emulsions, small and large unilamellar vesicles (SUVs and LUVs, respectively) and giant unilamellar vesicles (GUVs). The advantage of vesicles is that they are free-standing closed lipid bilayers with minimal or no contact to the support, no presence of residuals from their preparation, and they represent a very good reporter of membrane properties.

SUVs and LUVs, to which we will refer from now on as liposomes, are classical membrane models with straightforward preparation and characterization. Bulk experiments performed with populations of liposomes are inherently limited to the sampling of the whole population and the obtained results are averaged out over individual inhomogeneities. Yet, single-liposome assays are becoming increasingly popular^5^,^6^,^7^. On the other hand, cell-sized GUVs are observed and manipulated under the microscope as single objects and GUV imaging and manipulation have allowed the investigation of a wide range of membrane and vesicle properties^8^,^9^,^10^, which is otherwise very difficult or impossible to accomplish with small liposomes.

Many fluorescence-based measurements performed on the membrane require it to be immobile during sampling. This is the case of quantitative fluorescence techniques such as fluorescence recovery after photobleaching (FRAP) and fluorescence correlation spectroscopy (FCS), in which the examined region of interest (ROI) must be fixed during the measurement. An immobilized sample would also facilitate other imaging techniques, including fluorescence lifetime microscopy (FLIM) and three-dimensional reconstruction of GUVs with confocal microscopy as well as with super-resolution microscopy techniques. Very often, 3D imaging is limited to one vesicle half (rather than the whole vesicle surface) due to time constraints. The obtained images are often distorted or blurry due to small movements of the vesicle. Lateral displacements may lead to artifacts and, hence, result in data which are either useless or difficult to interpret.

Despite the need, only a few studies have attempted establishing vesicle immobilization. GUVs and liposomes can be immobilized at a functionalized surface through specific biotin-avidin binding^11^, but this approach leads to changes in composition of the adhering area^12^,^13^. Another possibility is to apply a magnetic or electric field to trap the vesicles^14^, but in this case, tension is imposed on the membrane. Optical traps or stretchers can also be employed, but one either needs a handle in the form of a particle attached to the membrane or else local heating may occur^15^, and both setups require sophisticated equipment. The latter applies also to vesicle trapping in microfluidic devices^16^. Alternatively, GUVs prepared on electrode wires remain attached to the electrodes^17^ but the vesicle is not isolated and typically connected by a thin tether to the electrode. An interesting option is to arrest vesicles inside a mesh of polymerizable molecules. Esquembre *et al.* reported efficient GUV immobilization on a mesh of porous silica glasses^18^. However, all measured parameters such as lipid order and molecular mobility were significantly altered by the support and larger GUVs were observed to collapse. In a similar approach, hydrogelators were used to immobilize proteo-GUVs^19^, but protein activity was shown to be reduced upon immobilization. Similarly, Tsumoto *et al.* used relatively high agarose concentrations to study morphological and permeability changes induced on embedded GUVs by adding membrane-active molecules^20^, but no detailed characterization of possible immobilization effects was shown.

In this work, we report a functional, efficient and simple vesicle immobilization method based on the thermal properties of agarose polymers. The vesicles were dispersed in fluid agarose, above the polymer melting temperature, and became readily immobilized when the dispersion cooled down to room temperature and agarose became a gel. The immobilization method proposed here is simple and fast to implement, does not require any special equipment, expensive chemicals or expertise in microfluidics design, and is potentially applicable in any laboratory.

## Results

Extracting quantitative information from experiments with GUVs is often challenging. In many applications, it is crucial that the GUVs remain immobile throughout the sampling time, which may span up to minutes. In a typical experiment, GUVs are dispersed in aqueous solutions and diffusive motion and convective flows lead to vesicle drift inside the observation chamber. These movements preclude or at best make these measurements difficult. To expand the range of routine biophysical applications of GUVs, we envisaged a simple albeit efficient immobilization method, based on the presence of agarose gel in the external vesicle solution.

Low-melting temperature agarose polymer (T_m_ ~ 62 °C, T_g_ ~ 26 °C) was used to immobilize GUVs and liposomes. Agarose forms a gel at room temperature and is fluid at temperatures above the melting temperature T_m_. It exhibits large hysteresis, becoming a gel again when the temperature is decreased below the gelation temperature T_g_. Vesicles and agarose were mixed while the polymer was still in the fluid state (around 35–40 °C) at 0.5% w/v agarose concentration if not mentioned otherwise. This concentration was chosen based on the best balance between immobilization efficiency and undesired morphological deformations (see below). After mixing, the sample was left for at least ten minutes at room temperature for agarose jellification. Interacting molecules were added to the sample before or after polymer jellification as further indicated for the given experiment (see sketch of the observation chamber in Fig. S1).

### GUVs are fully immobilized but unperturbed by the agarose gel

**Figure 1:**
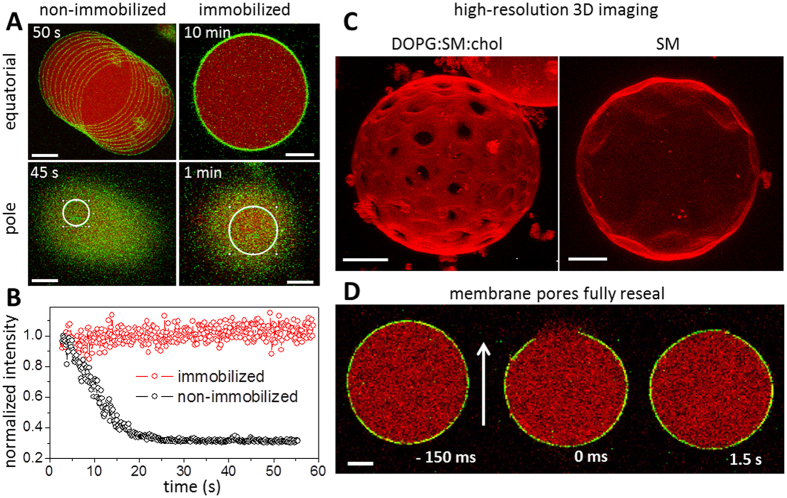
GUV immobilization efficiency. (**A**) Overlay of consecutive snapshots of POPC GUVs in the absence (left) and presence (right) of 0.5% w/v agarose gel in the external medium. Upper and lower images show confocal images of the equatorial cross sections (top) and the vesicle surface at the poles (bottom), respectively. The total measurement time is shown in the top corner of each picture. The membrane is labeled with 0.5 mol% of NBD-PE (false green color) and the vesicles encapsulate 2.5 μM SRB (false red color). (**B**) Fluorescence intensity of NBD-PE measured inside the ROIs indicated in A (white circle – proportional to the GUV size) as a function of time. (**C**) High-resolution 3D image reconstructions of GUVs immobilized in 0.5% w/v agarose. Left: GUV composed of DOPG:SM:chol (3:5:2–molar ratio) with 0.1 mol% of DiI C18. Ld and Lo domains are visible as bright and dark regions in the GUV membrane, respectively. Cross-sections (262 in total) were acquired at 512×512 pixels and 400 Hz scanning speed. Right: GUV made of made SM (in the gel phase) with 0.1 mol% DiI C18. Cross-sections (100) were acquired at 512×512 pixels and 400 Hz scanning speed. (**D**) Confocal snapshots of a GUV labeled with 0.5 mol% NBD-PE (green) dispersed in 0.5% w/v agarose and encapsulating 2.5 μM SRB (red). The images show the vesicle before and after membrane poration induced by an applied DC pulse (3 kV/cm, 150 μs). The pore region corresponds to the membrane discontinuity segment. Numbers correspond to time relative to the application of the pulse. The electric field direction is shown as an arrow. All scale bars correspond to 10 μm.

In a typical experiment and without any immobilization strategy (e.g., fixing or tethering to a surface or by means of optical trapping, micropipette manipulation or microfluidic posts), GUVs display micrometer-length lateral displacement during typical observation times (from several seconds to a few minutes). The drifting becomes even more pronounced in the presence of convective flows ensuing from the assembly of the observation chamber. An example of such GUV displacement is shown in Fig. 1A (upper-left image), in which consecutive snapshots of a free GUV taken every 5 s are overlaid in one image. In large contrast, when dispersed in 0.5% w/v agarose gel, vesicles are fully immobilized, displaying no visible lateral displacement at least within 10 min (Fig. 1A, upper-right image). Importantly, thermal shape fluctuations are not suppressed (see movie in the Supplementary Information), although possibly reduced. At low concentrations of agarose (up to 0.1% w/v), vesicles are still able to move, although much more slowly (a few μm/min), whereas at concentrations of 0.25–0.5% w/v agarose or higher, their lateral displacement is completely suppressed (see Fig. 1A). At high agarose concentrations (1% w/v agarose), deformed GUVs and GUVs with long and externally protruding lipid tubes were often observed (Fig. S2).

Immobilization efficiency was examined not only for the equatorial cross sections but also at the vesicle poles, since many fluorescence-based applications, such as FRAP and FCS, require observation of a selected membrane area (region of interest, ROI) imaged for long times. Figure 1A, lower-left image, shows overlay images of the upper pole of a non-immobilized GUV during the measurement time (45 s). The vesicle clearly displayed a drift. In contrast, immobilization efficiently preserves the vesicle position for at least several minutes (Fig. 1A, lower-right image). In both cases, ROIs (white circles with radii proportional to the GUV sizes) were selected at the GUV pole in the first imaging frame. Membrane fluorescence intensities within the ROIs (normalized to the first frames) are shown as a function of time in Fig. 1B for both cases. Whereas the fluorescence intensity remains constant for the immobilized vesicle (red data points), it varies significantly when the non-immobilized vesicle drifts during the measurement (black data points). This result demonstrates the efficiency of the immobilization method for performing quantitative measurements both at the GUV equator and the poles.

One of the obvious benefits of the immobilization strategy is to obtain high-resolution 3D images of GUVs. Such image reconstructions allow complete representation of the vesicle topology and are particularly important for imaging GUVs exhibiting phase separation. Figure 1C shows detailed reconstructed images of two GUVs immobilized in agarose. The image on the left shows a vesicle composed of DOPG:SM:chol (3:5:2–molar ratio) exhibiting Lo/Ld (liquid-ordered/liquid-disordered) phase separation. The image is a whole-vesicle 3D projection reconstructed from 262 slices. The acquisition took over 5 minutes, which would be very difficult to achieve with freely suspended GUVs. Domains as well as their shapes are clearly seen in the image, which contains no blurriness whatsoever. The image on the right in Fig. 1C was obtained from the upper hemisphere of a GUV made of SM, which exhibits facets characteristic of the gel phase. The surface topology can be clearly seen. Images with higher resolution demanding even longer sampling times could also be obtained.

One question that arises when immobilizing the vesicles is whether the jellification process leads to shrinking or rupturing of the larger ones due to possible mechanical strain. We thus examined the effect of immobilization on GUV stability and on membrane permeability. The size distributions of GUV populations in the presence and absence of agarose are nearly the same (see Fig. S3), showing that even large vesicles remain stable in the presence of agarose, in contrast to results with other immobilization protocols^18^. This observation indicates that jellification proceeds only in the solution around the vesicles, surrounding them in cages. Note that the jellification process does not change significantly the solution osmolarity (the molar concentration of 1% w/v agarose is less than 1.5 mM^21^). We assume that agarose is depleted from a thin region around the vesicles, allowing them to perform shape fluctuations (see online movie). Importantly, populations of GUVs encapsulating the aqueous probe sulforhodamine B (SRB) were monitored for at least 1 h. Permeable GUVs were never observed for hundreds of vesicles analyzed.

### Forcedly induced pores in immobilized vesicles fully reseal

GUVs have been used in a number of studies investigating pores in membranes, whether generated by pore-forming agents, see e.g.^22^,^23^,^24^,^25^,^26^, or induced externally, e.g. by electric fields^27^,^28^. We questioned the applicability of GUV immobilization for such studies. In particular, we investigated whether the resealing of large pores in the membrane would be prevented by the agarose gel as recently observed for GUVs containing agarose in their interior^29^. To induce the micron-sized pores (macropores), we exposed immobilized GUVs to strong electric pulses. The macropores fully resealed and membrane integrity was completely restored (see Figs 1D and S4). Leakage of the internal content occurred only during the time when the macropore was open (see Fig. S5). This finding is important, because it demonstrates that while pores in vesicles grown on hybrid films of agarose and lipids (following the protocol in ref. 30) do not reseal, GUVs immobilized post formation can be employed for studies with pore-forming molecules provided they do not interact with agarose (a detailed description of how agarose in the internal and external media affects vesicle deformation, poration and relaxation dynamics is presented in section S4 of the Supplementary Information). Indeed, we expect that while the dynamics of resealing of pores in the micrometer range is slowed down, the closure of nanometer-sized pores would not be affected. The results broadly validate that the mechanical confinement provided by agarose does not affect the vesicle structural integrity and does not hinder the complete resealing of membrane pores even in conditions where they were forcedly induced.

### Water-soluble molecules can freely diffuse through the agarose mesh

**Figure 2:**
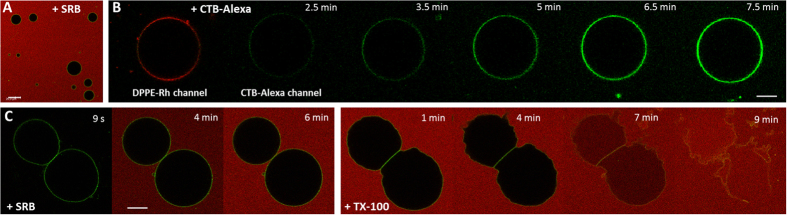
Diffusion of water-soluble molecules in the agarose gel. (**A**) Field with several immobilized GUVs (POPC with 0.5 mol% NBD-PE, green) obtained ~15 min after addition of an aliquot of SRB (red) to yield a final 2.5 μM concentration. Scale bar represents 50 μm. (**B**) Confocal microscopy images of an immobilized GUV (POPC with 1 mol% GM1 and 0.1 mol% DPPE-Rh, red channel) acquired after addition of CTB-Alexa (green channel) to yield a final 25 ng/mL concentration. The scale bar represents 10 μm. (**C**) Confocal microscopy images of two GUVs (POPC with 0.5 mol% NBD-PE, green). An aliquot of SRB (red) was added to the chamber with immobilized vesicles to yield a final 2.5 μM concentration. After establishing homogenous distribution of SRB in the chamber (6 min), an aliquot of TX-100 was added to the same sample to yield a final concentration of 1 mM (above its critical micelle concentration and therefore able to solubilize the membranes). The time stamp refers to the moment when SRB or TX-100 were added to the chamber with immobilized GUVs. The scale bar represents 20 μm.

If the agarose gel does not hinder the diffusion of water-soluble molecules and particles, our protocol could then be applicable to study their binding to the membrane. Thus, we attempted to determine whether molecules and particles added to the chamber after agarose jellification can freely diffuse through the agarose mesh and reach the immobilized vesicle. To test that, different molecules were added to the observation chamber after immobilization of GUVs (see Fig. S1 for a sketch): the inert and small aqueous dye SRB (0.6 kDa), the detergent TX-100 (0.6 kDa monomers forming ~90 kDa micelles above 0.3 mM), which solubilizes the membrane, and cholera toxin B labeled with Alexa 488 (CTB-Alexa), a pentameric protein (~57 kDa) that binds to GM1 gangliosides present on the membrane surface. In a first experiment, SRB was added to the chamber with already immobilized vesicles (see Fig. S1). After about 10 min, the SRB fluorescence was homogeneous throughout the chamber and the GUVs remained impermeable to the dye (Fig. 2A). In a second experiment, CTB-Alexa was added to the chamber with immobilized GUVs made of POPC doped with 1 mol% GM1 and 0.1 mol% DPPE-Rh. The protein CTB-Alexa was able to freely diffuse through the agarose gel and within less than 10 min reach the vesicles and bind to their surface (Figs 2B and S6). In a third experiment, the ability of TX-100 micelles (~5 nm) to reach and solubilize immobilized POPC GUVs was tested. Initially, SRB was added and in less than 10 min its distribution in the chamber was homogeneous (Figs 2C and S6). Afterwards, an aliquot of the detergent TX-100 was added and the same vesicles were followed over time. After a few minutes, the detergent reached the selected vesicles and solubilized them (the last snapshot of Fig. 2C shows the solubilization process, which eventually finishes with no remaining bilayer structures–image not shown). Area expansion due to incorporation of TX-100 into the bilayer as reported earlier^25^ could be observed here as well and was not significantly hindered by the agarose scaffold (see snapshots 1–4 min in Fig. 2C). The three types of experiments consistently demonstrate that water-soluble molecules and particles with sizes up to ~5 nm (corresponding to the size of the TX-100 micelles), with no specific affinity for agarose, can diffuse through the mesh and eventually reach the GUV surface even when externally added to the gel after immobilization of the GUVs. Thus, our protocol opens possibilities for applications where interactions of molecules and nanoparticles with the membrane (involving processes such as binding, pore formation and solubilization) can be directly studied on immobilized vesicles.

To explore more precisely the influence of the agarose gel on the diffusion of small molecules such as SRB, we used FCS to measure the diffusion coefficient (D) of SRB in the presence and absence of the agarose gel. For such molecules, FCS is preferred over FRAP since the former technique is more suitable to study fast moving objects. Fig. S7A shows a typical autocorrelation curve for SRB in solution with the corresponding fit and the diffusion coefficient values obtained in the presence and absence of 0.5% w/v agarose. The mean values obtained were D = 404 ± 25 μm^2^/s (no agarose) and D = 414 ± 10 μm^2^/s (in 0.5% w/v agarose), comparable to data in the literature^31^,^32^. Diffusion coefficient assessed with FCS probes short-range displacements (within the confocal volume). To probe long-range diffusion, FRAP measurements were performed with the aqueous probe carboxyfluorescein (CF, 0.4 kDa) in the presence and absence of 0.5% w/v agarose using a large photobleaching spot (20 μm diameter). The diffusion coefficients obtained in both cases are equal within the experimental error: D = 367 ± 60 and 417 ± 75 μm^2^/s for CF in water and in agarose gel, respectively, as shown in Fig. S7B. These results corroborate our finding that the diffusion of small molecules is not affected by the agarose mesh at the agarose concentration used here, at least up to the micrometer range. In fact, previous results showed unhindered diffusion of macromolecules (<10 nm) in the presence of even higher agarose concentrations (1.5% w/v agarose)^33^. Therefore, the immobilization strategy shown here can be used to study the effect of various water-soluble (macro)molecules on membrane properties.

### The presence of the agarose gel does not affect lipid diffusion

**Figure 3:**
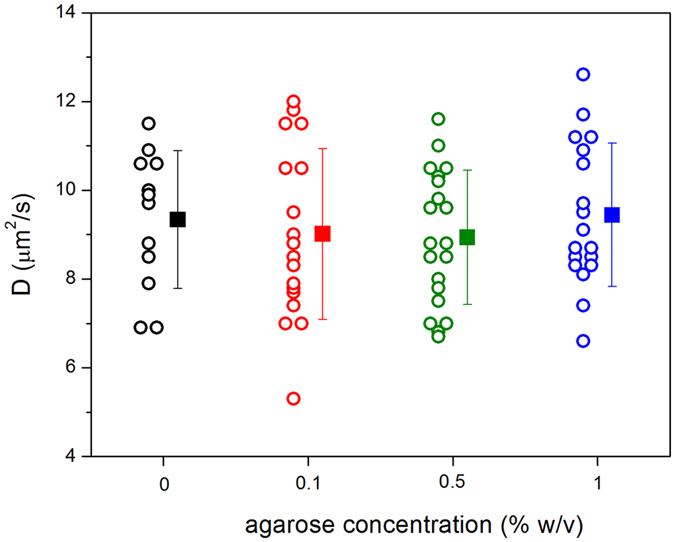
Lipid diffusion coefficient D as a function of agarose concentration in the vesicle exterior. Each point represents a single FRAP measurement on an individual GUV and the mean values with standard deviation for every agarose concentration are indicated. The GUVs were composed of POPC with 1 mol% NBD-PE.

The diffusion coefficient of NBE-PE lipids in immobilized GUVs was measured with FRAP for increasing agarose concentrations and compared with data collected on agarose-free (non-immobilized) GUVs. Photobleaching was performed on the vesicle poles and diffusion during photobleaching was taken into account in the determination of the diffusion coefficient (see Experimental Section and ref. 34). FRAP measurements on non-immobilized vesicles is challenging and a large number of data (>50%) had to be discarded due to significant GUV drift during measurements or artifacts in the recovery curves. In contrast, FRAP is very easy to perform on agarose-immobilized GUVs. Figure 3 shows values of the diffusion coefficients obtained on POPC GUVs at increasing agarose concentration. The mean values measured for each agarose concentration were D = 9.3 ± 1.6 (0%), 9.0 ± 1.9 (0.1%), 8.9 ± 1.5 (0.5%) and 9.4 ± 1.6 (1%) μm^2^/s. These values are in the upper range of values reported in the literature (3–10 μm^2^/s) from different model systems using different techniques^1^,^35^,^36^,^37^,^38^,^39^. However, most of the reported FRAP data^35^,^38^ were analyzed without correcting for diffusion during photobleaching, and are probably underestimated, as discussed in ref. 34. More importantly, the above results show that lipid diffusion in the GUVs is unhindered and agarose has no effect on lipid lateral mobility even at relatively high agarose concentrations.

### Applying GUV immobilization to measure the mobility of lipids in different conditions

Above, we demonstrated that vesicle immobilization in agarose allows facile imaging and can be potentially used for precise quantification of membrane properties. The applications that we will now consider address the role of membrane phase state and charge on lipid mobility.

**Figure 4:**
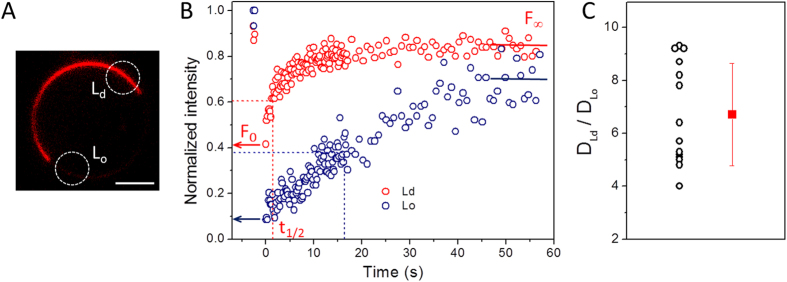
GUV immobilization allows quantification of lipid lateral mobility in phase separated membranes. (**A**) Confocal cross section of a DOPG:SM:chol (3:5:2–molar ratio) GUV immobilized in 0.5% w/v agarose. The membrane is labeled with 0.1 mol% of DiI C18. Ld and Lo domains are visible as bright and weakly fluorescent regions in the GUV membrane, respectively. Scale bar: 10 μm. (**B**) FRAP recovery data for Ld (red) and Lo (blue) domains performed on the ROIs (marked with dashed white circles in (**A**). F_0_, t_1/2_ and F_∞_ are the initial fluorescence after photobleaching, the half-time of recovery and the fluorescence after recovery, respectively. (**C**) Ratio of half-time of recovery t_1/2_ for the Lo and Ld domains obtained on the same vesicle. Each point represents a single GUV.

Lipid diffusion is strongly influenced by the phase state of the membrane. Of special biological relevance are liquid phases with properties modulated by the presence of cholesterol. As shown in Fig. 1C (left), ternary lipid mixtures can exhibit Lo/Ld phase coexistence, relevant for studies on raft-like systems. It is well known that lipid mobility is strongly reduced in the Lo phase compared to the Ld phase^40^. GUV immobilization not only enables precise quantification of the lipid lateral mobility in each of the phases but allows such measurements to be performed on the same vesicle. Such experiments are challenging to conduct on freely suspended GUVs. Here, FRAP measurements were performed in the Lo and Ld phases of fully phase-separated GUVs made of DOPG:SM:chol 3:5:2 and immobilized in agarose. For such experiments, a photostable dye with preferential partitioning in one phase but present in both phases, is required. DiI C18 fulfills these requirements and is known to prefer the Ld phase^41^, which appears brighter. FRAP was performed in the vesicle equator (Fig. 4A) instead of at the pole regions, since each domain was not necessarily present at the vesicle poles. Recovery data for Ld and Lo phases performed in the same GUV (with domains equatorially opposite to each other to avoid polarization effects) are shown in Fig. 4B, with half-time (t_1/2_) of full recovery being 1.5 and 17.7 s, respectively. Since the geometry of the bleached area in the equator is different from that on the vesicle poles, the analysis discussed in eqs. 1 and 2 to extract the diffusion coefficient from the recovery curves cannot be directly applied here. Therefore, Fig. 4C shows the ratio of t_1/2_ obtained for the Lo and Ld phases in the same vesicle, which should be proportional to the ratio between the diffusion coefficients of the two phases, D_Ld_/D_Lo_. The scatter in the data probably reflects the scatter in membrane composition within the batch resulting from the preparation method^42^. The results show that, for this membrane composition, diffusion in the Ld phase is ~7 times faster than that in the Lo phase, similarly to data reported for neutral GUVs^40^,^43^. Note that the value of the ratio will depend on the exact vesicle composition, where the domain compositions are defined by the tie line. To summarize, we demonstrate the utility of the agarose-based immobilization method to perform FRAP measurements on different domains in the very same phase-separated GUV.

**Figure 5:**
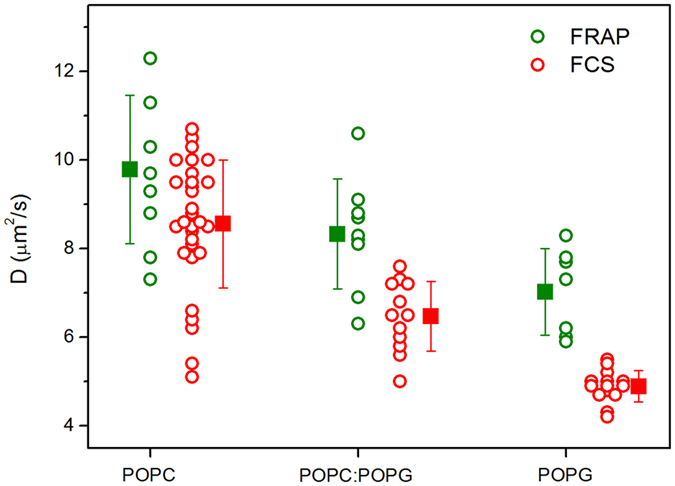
Diffusion coefficient D measured for 1 mol% NBD-PE (FRAP, green) or 0.002 mol% DiI C18 (FCS, red) in neutral (pure POPC) and charged (POPC:POPG 1:1 and pure POPG) GUVs immobilized in 0.5% w/v agarose. Each point represents a measurement on different GUV (for FRAP, one measurement per GUV was performed whereas for FCS, up to three measurements per vesicle were performed). The squares are mean values with standard deviations.

Next we examined the role of membrane charge on the lipid diffusion of homogeneous fluid membranes. The general understanding in the literature is that lipid mobility is reduced in charged membranes^35^,^36^,^44^. The diffusion coefficient experiments here were performed on the poles of immobilized GUVs composed of pure POPC, POPC:POPG 1:1 (molar ratio) and pure POPG. The effect of membrane charge on the diffusion coefficient was probed using both FRAP and FCS and the results are summarized in Fig. 5. The measured diffusion coefficient decreases as the fraction of anionic lipids increases. This trend is observed both with FRAP and FCS measurements. FRAP yields slightly higher values presumably because of the different probe used and because of differences in the measurement techniques^38^. The consistent trends indicate that reduction in lipid mobility is a true behavior rather than an experimental bias. In Table S1, we have compiled values of diffusion coefficients measured with both techniques.

### Small liposomes can also be immobilized by the agarose gel

Small liposomes (~100 nm diameter) are extensively used as biomimetic membrane model in a wide variety of applications. Very often, in studies investigating binding of molecules to the membrane or bilayer-assisted reactions, liposome clustering or aggregation occurs, see e.g. refs 45,46. Approaches have been reported by which aggregation may be avoided, for example via coating of the liposomes with steric inhibitors, such as polyethylene glycol^47^. This approach, however, might hinder the investigated interaction. Dispersion of liposomes in agarose gel could be used as another effective means to suppress aggregation by significantly reducing liposome mobility and hence the likelihood of contact between liposomes.

**Figure 6:**
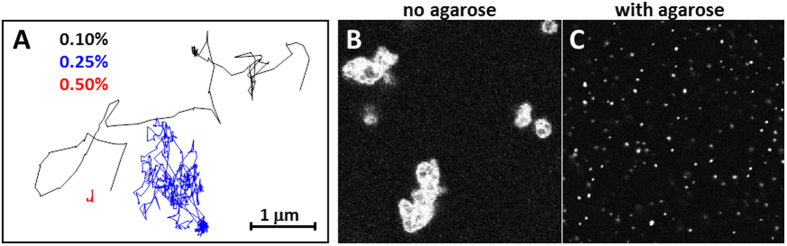
Immobilization of LUVs in agarose gels. (**A**) Trajectories of single fluorescently labeled 100-nm liposomes (DOPE:DOTAP:DPPE-Rh 1:1:0.1 mol) tracked during 30 s (150 ms/frame) in the presence of 0.1 (black), 0.25 (blue) and 0.5 (red) % w/v agarose. (**B**) Atg8-PE induced liposome (DOPE:POPC:chol:POPG 63.6:13.6:13.6:9.1) aggregates in the absence of agarose. (**C**) Liposome aggregation by Atg8-PE is prevented by addition of 0.5% w/v agarose. Individual protein-labeled liposomes are clearly observed. In panels B and C, the fluorescence signal is from fluorescently labeled Atg8. The picture dimensions are 110 μm × 110 μm.

We used the agarose gel method to test whether small liposomes could also be efficiently immobilized. A suspension of fluorescently labeled liposomes (at total lipid concentration of 20 μM) was investigated at increasing concentrations of agarose. To quantify the mobility of the liposomes, their trajectories were recorded for 30 seconds using confocal microscopy. In the absence of agarose, the liposomes could not be individually tracked due to their fast movement. At 0.1% w/v agarose, individual liposomes could be followed and their movements spanned distances of several micrometers (black data in Fig. 6A). Liposome mobility was significantly reduced when the polymer concentration was increased to 0.25% w/v (blue data) and was virtually suppressed upon further increase to 0.5% w/v agarose (red data). Another approach to probe liposome mobility based on detection of moving particles inside a ROI is shown in Fig. S8. The liposomes were immobilized in most areas of the observation chamber at 0.5% w/v agarose concentration, but they were still able to display μm-length displacement in certain regions (mostly at the chamber edges), probably due to inhomogeneous mixing. Nonetheless, these results demonstrate that small liposomes can be completely immobilized inside the gel at similar agarose concentration as that used for GUV immobilization. This finding opens up new possibilities to perform quantitative measurements on small particles or liposomes as demonstrated below.

As an example, we investigated a protein-lipid reaction that is usually accompanied by vesicle aggregation. The autophagy-related protein Atg8 is known to covalently bind to phosphatidylethanolamine (PE) in eukaryotic membranes in a complex conjugation reaction involving two additional proteins, Atg3 and Atg7, and ATP^48^. Membranes containing Atg8-PE were shown to aggregate *in vivo* and *in vitro*^48^,^49^,^50^,^51^. Thus, hindering aggregation of liposomes with Atg8-PE is important for quantifying the protein coverage on membranes. Here, we investigated the influence of the agarose gel on the complex biochemical reaction of Atg8-PE conjugation and the ability of agarose to prevent liposome aggregation.

Atg8 was fluorescently labeled^48^,^49^ and added, together with Atg3, Atg7 and ATP, to non-labeled freely-suspended and agarose-immobilized PE-containing liposomes. As expected, the Atg8-PE binding reaction induced strong liposome aggregation in agarose-free solution (Fig. 6B). In sharp contrast, carrying out the same reaction with immobilized liposomes does not lead to vesicle aggregation and single individual fluorescent spots corresponding to protein-bound liposomes are clearly seen as diffraction-limited spots (Fig. 6C). The results also show that the proteins are able to diffuse through the agarose meshwork as shown for other molecules in Fig. 2. Our results demonstrate that the complex biochemical reaction involving Atg8, Atg3, Atg7 and ATP occurred on the liposome surface even in the presence of the agarose mesh. Moreover, liposome immobilization effectively prevented vesicle aggregation. In conclusion, the agarose immobilization method can be applied to any liposomal system and enables users to perform quantitative single-liposome experiments in a simple way.

## Concluding Remarks

In this work, we developed a simple method to efficiently immobilize lipid vesicles of various sizes, from 100 nm to 100 μm. The approach is based on confining the vesicles by agarose gel in the external medium. In the conditions studied here, giant vesicles are fully immobilized for times much longer (several minutes) than conventionally probed in experiments. Immobilization does not damage the vesicles and membrane integrity is restored even after the vesicles are exposed to drastic perturbation such as electroporation. Molecules, proteins and nanoparticles (with sizes below or equal to 5 nm) added after vesicle immobilization can freely diffuse through the mesh, reach the vesicles and interact with the membrane in the same way as in the absence of agarose. Importantly, the presence of the agarose gel has no detectable effect on the lateral diffusion of lipids. We applied the method to perform quantitative measurements, which are challenging and very difficult to establish without an immobilization strategy. As a proof of principle, we showed that the lipid diffusion coefficient is strongly dependent on membrane phase state and that it is reduced at increasing membrane charge density in fluid membranes.

As demonstrated in this work, the overall membrane structure and its permeability and fluidity are preserved after immobilization. However, a word of caution should be added: for some GUV-based experimental approaches, which probe the morphological responses of vesicles, such as electrodeformation^29^,^52^ and induction of membrane curvature^53^, immobilization might lead to adverse effects which should be carefully considered before employing this approach. For example, even though immobilized GUVs with excess area display visible shape fluctuations (see supplementary movie), the influence of agarose on the membrane bending stiffness using fluctuation spectroscopy (see e.g. ref. 54) was not quantified here because of potential interference of the gel on the vesicle contour detection.

The detailed internal structure of the agarose polymer is not known, but it is expected to form compartments of different sizes depending on a number of factors such as temperature, T_m_ of the used agarose type, agarose concentration and cooling speed. FCS measurements of diffusion coefficient of small probes inside a 1.5% w/v low T_m_ agarose yields pore size of ~70 nm and show anomalous diffusion for particles above 10 nm in size^33^. Estimates from absorbance measurements for low T_m_ agarose at 1% w/v and room temperature yield large pore sizes ~600 nm^55^. Based on our data, small molecules such as CF (0.4 kDa) and SRB (0.6 kDa), the proteins CTB, Atg3, Atg7 and Atg8 (13.6–71 kDa) and TX-100 micelles (~90 kDa; ~5 nm) are able to diffuse through the mesh. On the other hand, 100 nm liposomes are virtually immobilized in the presence of 0.5% w/v agarose. Therefore, we can conclude that at 0.5% w/v agarose, the compartment size should be smaller than 100 nm. This mesh size enables complete immobilization of micro and large structures such as liposomes and GUVs whereas the diffusional mobility of small molecules, proteins and micelles is preserved. This opens the possibility to study the interaction of various macromolecules and nanoparticles with the membrane of immobilized GUVs. We also expect that the immobilization strategy will allow performing permeabilization studies such as in refs 56,57.

We envision the agarose immobilization method to find a broad application as it allows long term imaging of vesicles without compromising the membrane structural integrity. Apart from the applications reported here, state-of-the-art super-resolution microscopy techniques, such as STED, STED-FCS and PALM/STORM microscopy^58^,^59^, will also profit from the agarose immobilization method, since one requires a reliable way of immobilization to achieve the stated resolution precision in the 20–30 nm range. In summary, the agarose immobilization method described here allows detailed quantitative investigation of biophysical membrane properties of giant and small vesicles with unprecedented simplicity.

## Experimental Section

### Materials

The lipids 1-palmitoyl-2-oleoyl-sn-glycero-3-phosphocholine (POPC), 1-palmitoyl-2-oleoyl-sn-glycero-3-phospho-(1′-rac-glycerol), sodium salt (POPG), 1,2-dioleoyl-sn-glycero-3-phosphoethanolamine (DOPE), 1,2-dioleoyl-3-trimethylammonium-propane, chloride salt (DOTAP), egg (chicken) sphingomyelin (SM), Ganglioside (Ovine Brain) (GM1) and cholesterol and the fluorescent probes 1-palmitoyl-2-6-[(7-nitro-2-1,3-benzoxadiazol-4-yl)amino]hexanoyl}-sn-glycero-3-phosphoethanolamine (NBD-PE) and 1,2-dipalmitoyl-sn-glycero-3-phosphoethanolamine-N-(lissamine rhodamine B sulfonyl) (DPPE-Rh) were purchased from Avanti Polar Lipids (Alabaster, AL). The fluorescent probe 1,1′-Dioctadecyl-3,3,3′,3′-Tetramethylindocarbocyanine Perchlorate (DiI C18) was obtained from Life Technologies (Carlsbad, CA). Low-melting temperature agarose was obtained from Fischer Scientifics (Walthan, MA) and Cholera Toxin Subunit B (Recombinant) Alexa Fluor 488 Conjugate (CTB-Alexa) was purchased from Invitrogen (Carlsbad, CA). The fluorescent dye sulforhodamine B (SRB), carboxyfluorescein (CF), the detergent Triton X-100 (TX-100) and all other chemicals were purchased from Sigma Aldrich (St. Louis, MO). All chemicals were used without further purification. Milli-Q water was used throughout the work.

### Preparation of giant and large unilamellar vesicles

GUVs were produced by the electroformation method^60^ with minor modifications. In brief, 5–8 μL of a given 3 mM lipid stock solution in chloroform were homogeneously spread on a pair of conductive ITO glasses and dried for 5 minutes under N_2_ stream. A 2 mm-thick Teflon spacer was used to create a chamber between the glasses, which was then sealed and filled with a 0.2 M sucrose solution. The chamber was connected to a function generator and an AC field (1.2 V, 10 Hz) was applied at room temperature for 1 h to swell the GUVs. Only when containing agarose inside, vesicles were grown at 70 °C. When fluorescent lipids were included, GUV growth was performed in the dark. Afterwards, the GUVs were diluted ~7 fold in isoosmolar glucose solution containing or not fluid agarose. For the phase-separated membrane composition and pure SM, lipid hydration was performed at 60 °C and with 50 mM sucrose, 2 mM HEPES and 1 mM EDTA, and the vesicles were then dispersed in hypotonic 40 mM glucose solution.

Liposomes were prepared by extrusion at room temperature. A chloroform solution of DOTAP:DOPE:DPPE-Rh (1:1:0.1 molar ratio) was placed in a round-bottom test tube. The chloroform was evaporated with a N_2_ stream and the test tubes were further dried under vacuum for 2 h. The lipid film was then hydrated with a 0.2 M sucrose solution to yield a final 2 mM lipid concentration and vigorously vortexed for 2–5 minutes. The multilamellar vesicles obtained were subjected to 11 cycles of extrusion through a 100 nm pore diameter polycarbonate membrane (Whatman, Maidstone, UK). To test liposome mobility as a function of agarose concentration, the liposome dispersion was diluted to 20 μM. In all cases, experiments were performed at room temperature. The liposomes conjugated to autophagy-related proteins were prepared by freeze-thaw cycling (see next section for details).

### Interaction of Atg8 with liposomes

The autophagy-related proteins Atg8, Atg7 and Atg3 were purified and Atg8 was labeled with Alexa 488 as reported previously^48^,^49^. Liposomes composed of 63.6 mol% DOPE, 13.6 mol% POPC, 13.6 mol% cholesterol and 9.1 mol% POPG were suspended in 50 mM Tris-HCl pH 8, 10 mM NaCl, 0.1 mM MgCl_2_. The agarose was dissolved in the same buffer at a final concentration of 1% w/v. The proteins were mixed with the liposomes in the presence of ATP (1.2 μM Atg8, 0.2 μM Atg7, 0.2 μM Atg3, 0.7 mM lipid, 2 mM ATP) and then diluted 1:1 with 1% w/v agarose buffer in the fluid state. The samples were placed between cover slips sealed by silicon spacers and incubated for 1 min on ice to induce gelation of the agarose. For the Atg8 conjugation reaction, the samples were protected from light during incubation for 100 min at 22 °C and then analyzed by confocal microscopy.

### Application of DC pulses to GUVs

To examine vesicle deformability and monitor the effect of agarose on closure of pores in the membrane, GUVs containing agarose either internally or externally were exposed to an electric pulse applied in an electrofusion chamber (Eppendorf, Germany) with parallel cylindrical electrodes (92 μm radius) spaced at 500 μm. A single DC pulse of 150 V field strength and 150 μs duration was applied for all cases. Vesicle imaging was carried out using a Zeiss Axio Observer.D1 microscope equipped with a sCMOS camera (pco.edge, PCO AG, Kelheim, Germany). The vesicles were observed either with a 20x (NA 0.5) or a 40x (NA 0.6) objective in the phase contrast or epifluorescence mode. For fluorescence microscopy images, excitation (540–553 nm) and emission (575–640 nm) filters were used.

### Confocal Microscopy

Confocal microscopy imaging, FRAP and FCS were performed on a Leica TCS SP5 (Wetzlar, Germany) with a 40x (0.75 NA) or 63x (1.2 NA) water immersion objectives and 1 Airy unit. NBD-PE, CTB-Alexa, CF and Alexa488-labeled Atg8 were excited with an argon laser at 488 nm and SRB, DiI C18 and DPPE-Rh with a diode-pumped solid-state laser at 561 nm. The emission signals were collected in the 490–545 nm and 575–630 nm bands, respectively. Image quantification was performed with the Leica software (Leica application Suite) or ImageJ. For liposome tracking, a spot tracker plug-in from ImageJ was used as in ref. 61.

### Fluorescence recovery after photobleaching (FRAP)

For FRAP measurements on membranes, the GUVs contained the fluorescent probes NBD-PE (1 mol%) or DiI C18 (0.1 mol%). Images (296 × 296 pixels) were recorded in the bidirectional scan mode at 1000 Hz and pinhole at 1 Airy unit. Three images at attenuated laser intensity (below 5%) were taken before photobleaching. Photobleaching was performed using the argon 488 nm (for NBD) or the 561 nm laser (for DiI C18) at maximum intensity for 720 ms (4 frames) through a circular ROI of nominal radius r_n_ = 2.5 or 5 μm. The laser was then switched back to attenuated intensity and the recovery images were recorded for several seconds. If not explicitly mentioned, photobleaching was performed on the upper or lower GUV surface. For FRAP of the aqueous probe CF, a concentrated CF solution was first prepared in ethanol (200 mM) and then diluted in water or in 0.5% w/v agarose to give a final working concentration of 0.2 μM CF. Images (296 × 296 pixels) were recorded in the bidirectional scan mode at 1400 Hz and pinhole at 1 Airy unit. Ten pre-bleach images (98 ms/frame) at attenuated intensity (10% with the 488 nm laser line) were taken before and five bleaching iterations (0.49 s) using the 458, 476, 488 and 496 laser lines at maximum intensity to bleach CF, with a nominal radius r_n_ = 10 mm. After bleaching, excitation at 488 nm was switched back to attenuated intensity.

Several ways to extract the diffusion coefficient D from FRAP recovery curves have been reported in the literature^62^,^63^,^64^. Here, the data were analyzed according to a simplified equation considering molecular diffusion during photobleaching^34^, hence reducing error. The diffusion coefficient D is given as:


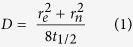


where r_e_ and r_n_ are the effective and the nominal (i.e., user-defined) bleaching radii and t_1/2_ is the half-time of fluorescence recovery (i.e., the time to reach F_1/2_ = (F_o_ + F_∞_)/2, where F_o_ and F_∞_ are the fluorescence intensity in the first post-bleach image and after full recovery, respectively; see Fig. S9). To obtain the effective bleaching radius r_e_, the fluorescence intensity line profile f(x) through the center of the bleaching spot in the first post-bleaching image was fitted with the expression


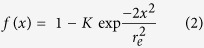


where *K* is the bleaching depth. Determination of r_e_ in our setup is shown in the Supplementary Information (Fig. S9) and the values used were r_e_ = 7 (for r_n_ = 2.5 μm) and 9 (for r_n_ = 5 μm) μm. A typical recovery curve is also shown (Fig. S9).

### Fluorescence correlation spectroscopy (FCS)

FCS measurements were performed with water-soluble (SRB) and membrane-embedded (DiI C18) fluorescence probes with a 63x (1.2 NA) water immersion objective. The sample was excited at 561 nm and fluorescence emission was collected in the band 607–683 nm using a filter cube. Photon counting was accomplished by avalanche photodiodes (Leica, Wetzlar, Germany) and time correlations were calculated at a sampling frequency of 200 kHz. Initially, to determine the geometry of the FCS volume, the correlation curves obtained from 10 nM SRB in 0.2 M sucrose were fitted globally with a 1-component 3D diffusion model with a fixed diffusion time of D = 410 μm^2^/s^31^,^32^:


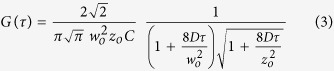


where C is the average molecule concentration in the FCS volume and τ the average time of the molecule in the focus volume. From the fit, the lateral and axial lengths of the FCS volume were obtained: w_0_ ≈ 0.29 μm and z_0_ ≈ 2.31 μm, respectively. Then, measurements of 10 nM SRB inside and outside immobilized GUVs were performed and eq. 3 was used to extract the diffusion coefficient D in the presence and absence of the agarose gel, respectively. The dye was present in the (fluid) glucose solution with agarose in which the GUVs were dispersed. At least 3 measurements were obtained per data point.

For FCS performed on GUV membranes, DiI C18 (0.002 mol%) was also excited using the 561 line. The obtained correlation curves were fitted using a 1-component 2D diffusion model:


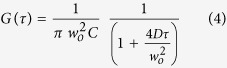


The confocal volume was positioned either at the top or at the bottom of the vesicles to yield the maximum photon count. To minimize artifacts, the average number of fluorescent molecules was plotted as a histogram and only the measurements that showed roughly the same apparent dye concentration (Fig. S10) were considered. This procedure does not alter the general trends but only reduces the measurement error. After correction, at least eight vesicles per population were considered.

## Additional Information

**How to cite this article**: Lira, R. B. *et al.* Posing for a picture: vesicle immobilization in agarose gel. *Sci. Rep.* **6**, 25254; doi: 10.1038/srep25254 (2016).

## Supplementary Material

Supplementary Information

Supplementary movie
