# Supplementary material for: Posing for a picture: vesicle immobilization in agarose gel
Source: Sci Rep. 2016 May 3;6:25254. doi: 10.1038/srep25254 (PMC4853705; doi:10.1038/srep25254)
Supplement: Supplementary Information [file srep25254-s1.pdf]

Supplementary information

Posing for a picture: vesicle immobilization in agarose gel

Rafael B. Lira, Jan Steinkühler, Roland L. Knorr, Rumiana Dimova, Karin A. Riske

Online movie. Movie of a fluctuating POPC GUV (labeled with 0.1 mol% DiI C18) obtained with confocal microscopy. The GUVs were dispersed in 0.2 M glucose with 0.5% w/v agarose. After jellification of agarose, the chamber was opened and the gel was exposed to a hyperosmotic solution (0.3 M glucose) inducing deflation of the immobilized vesicles. Thermal shape fluctuations are clearly detected in the GUV and are not suppressed by the agarose mesh. Additionally, no attachments to the agarose cage are observed. The time is indicated in the movie. The vesicle size is approximately 8 μm.

S1. Observation chamber with agarose-immobilized GUVs.

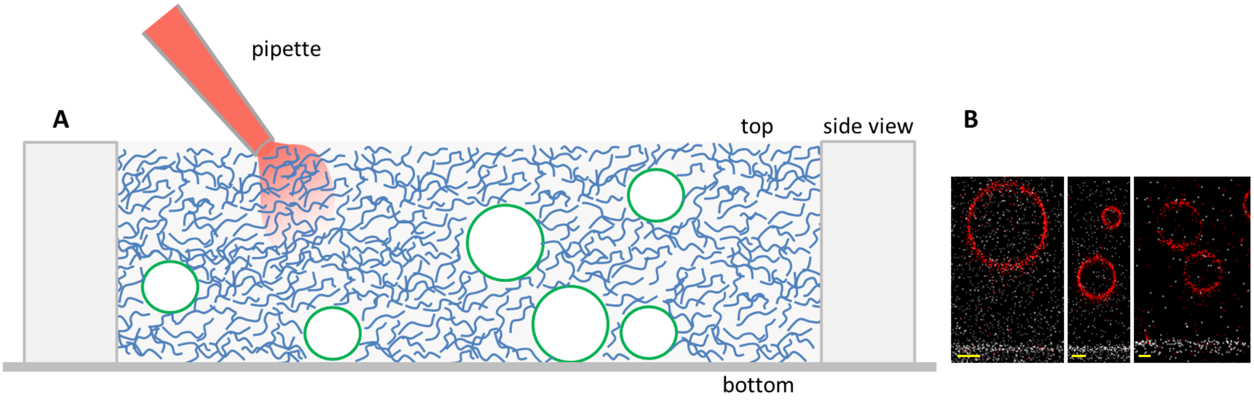

Figure S1. A) Sketch of the side view of the observation chamber. The GUVs are represented as green circles and agarose is shown in blue. GUVs were added with fluid agarose at high temperature (35-40 ^{o}C). After ~ 10 min and cooling to room temperature the agarose becomes a gel and GUVs get immobilized. Larger GUVs typically reached the chamber bottom before immobilization, but many GUVs were trapped in the agarose mesh before having sedimented to the chamber bottom. The chamber is closed by a glass slide from above. When the effect of molecules added post gelation is investigated, the glass slide is removed and the solution pipetted directly on the gel. After adding an aliquot of the investigated molecule (e.g. SRB, CTB-Alexa, TX-100), the chamber is reclosed with a cover slip and observation started. Alternatively, the molecule is added together with fluid agarose and the GUVs. B) Confocal microscopy images showing different cross sections (xz plane) of a chamber with POPC GUVs (labeled with 0.1 mol% DPPE-Rh) immobilized before reaching the bottom (white region below, observed under transmitted light). The scale bars represent 10 μm.

S2. Tubulation and deformation of a GUV in the presence of 1% w/v agarose.

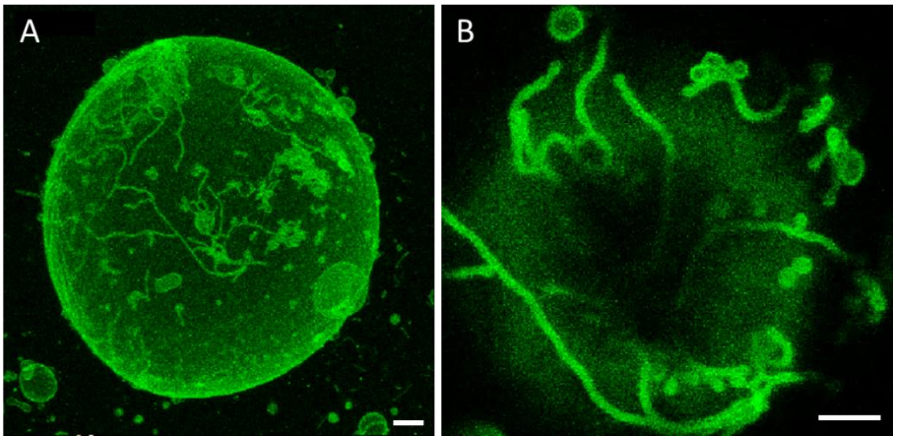

Figure S2. A) Three-dimensional reconstruction of a POPC GUV immobilized in 1% (w/v) agarose. Note that the vesicle is non-spherical and slightly deformed by the agarose gel. B) Image of the upper surface of the GUV shown in A. The formed tubes are always directed to the outside. Scale bars: 5 μm.

S3. Size distribution of GUVs in the presence and absence of agarose.

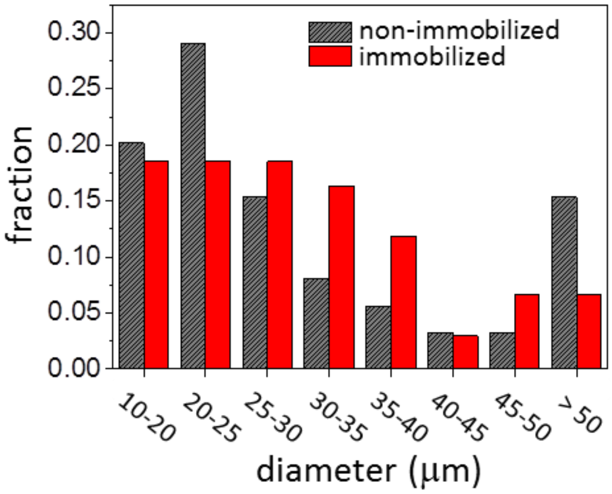

Figure S3. Vesicle size distributions from two typical batches of POPC GUVs in the presence (red, n = 118) and absence (black, n = 111) of agarose.

S4. Mechanical effects of agarose on GUVs

Recently, we showed that residual agarose present inside GUVs grown on hybrid films of agarose and lipids (following the protocol in Ref. [1]) significantly affects vesicle relaxation dynamics and membrane pore lifetime [2]. In the experiments shown here, where agarose was present exclusively outside, no adverse effects on vesicle size and stability were observed (Figure S3). In order to compare GUV mechanics in both conditions of agarose present exclusively in the vesicle exterior or interior, we followed the GUV response to electric fields. We applied a single and strong DC electric pulse to induce vesicle deformation and poration. In agarose-free aqueous solution, strong DC pulses deform and porate GUVs, after which the vesicle initial shape is restored with a characteristic relaxation time τ_{relax} and the formed macropores close with pore lifetime t_{pore} [3]. Deformation is expressed as the aspect ratio of the vesicle semi-axes, a/b (see the inset in Figure S4). A typical GUV deformation/poration relaxation dynamic in agarose-free solution upon pulse application is shown in Figure S6 (black circles). Vesicle relaxation time for this particular GUV is τ_{relax} ~ 20 ms. The horizontal line denotes the time period in which pores are optically detected, i.e. t_{pore}; in this case t_{pore} ~ 40 ms. Both values are in agreement with previous data [2,3] . After pore closure, the vesicle integrity is restored. For experiments with agarose inside, GUVs were grown with a sucrose solution containing 0.5 % w/v fluid agarose (at a temperature above agarose T_m) and then dispersed in glucose solution. At room temperature, all vesicles in the batch displayed an internal gel-like polymer mesh (see Figure S5A) and membrane fluctuations were largely suppressed. When a strong pulse is applied to GUVs encapsulating agarose, vesicle deformation is inhibited (a/b ~ 1, red triangles in Figure S4), very large pores open and the polymer mesh is expelled (Figures S5A and S5B). The formed pore never reseals due to the physical obstruction induced by the mesh (Figures S5A and S5B), a process reported earlier [2].

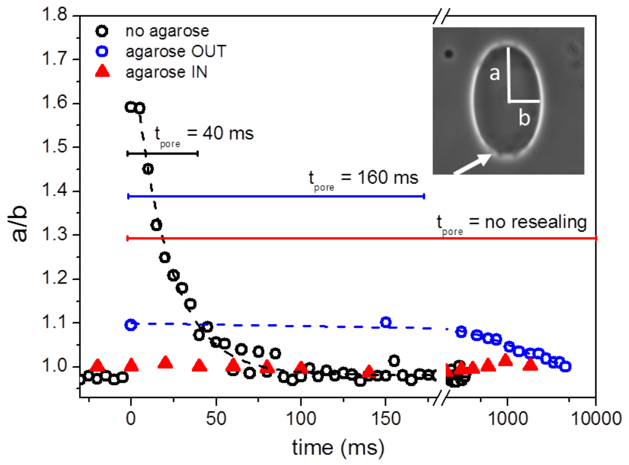

Figure S4. Vesicle aspect ratio a/b during electroporation for a GUV in agarose-free aqueous solution (open black circles), immobilized in agarose (open blue circles) and encapsulating agarose (solid red triangle). Dashed lines correspond to the fits using an exponential function with τ_{relax} as decay time. Horizontal lines indicate the period within which macropores are detected (corresponding to the pore lifetime t_{pore}). Inset shows maximal deformation of a GUV (aqueous solution) and its semi-axes a and b. The arrow points to the formed macropores.

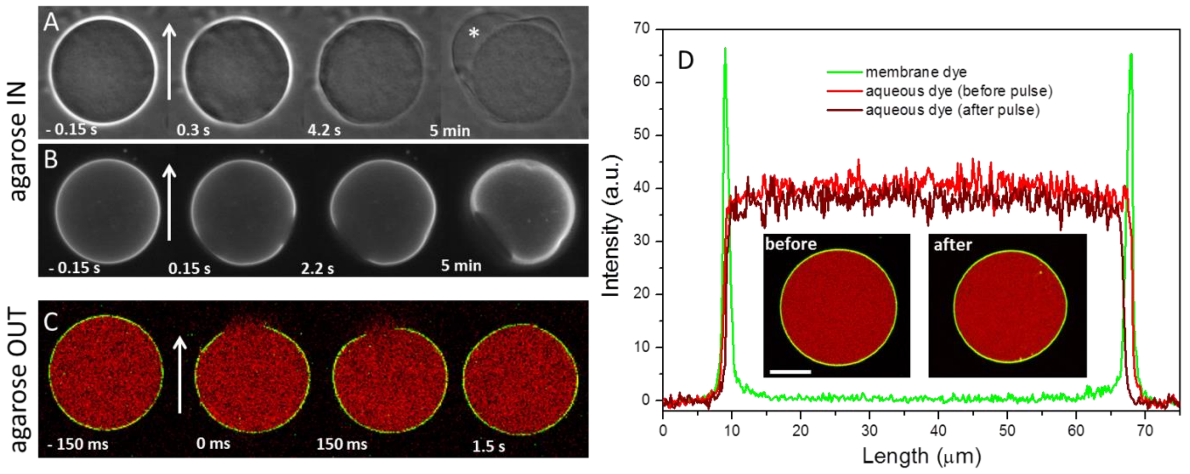

Figure S5. Effect of 0.5 % w/v agarose inside or outside GUVs exposed to electric pulses (3 kV/cm, 150 μs). A), B) Sequences of two GUVs encapsulating agarose during application of a DC pulse, observed by phase contrast (A) and epifluorescence (B). The asterisk in A indicates the remaining GUV membrane. The spherical object next to it is the agarose mesh located inside the GUV before poration. The diameters of the initial GUVs in A and B are about 40 μm. C) Confocal snapshots of a GUV dispersed in agarose and encapsulating 2.5 μM sulforhodamine B before and after application of a DC pulse. The membrane is labeled with 0.5 mol% NBD-PE. Numbers correspond to time relative to the application of the pulse. The electric field direction is shown as an arrow. D) Fluorescence intensity of the membrane (green) and of sulforhodamine B (red) across the vesicle (same as in panel C and Figure 2C in the main text) before (light red) and 1 min after (dark red) pulse application, measured on the corresponding confocal images shown as insets. Bars: 20 μm.

We next examined the response of immobilized GUVs (agarose only outside) to electric pulses. The vesicles contained encapsulated sulforhodamine B to allow detecting leakage through macropores and possibly long-lasting submicron pores, if present. As evidenced in Figure S5C, strong pulses lead to membrane poration (see interrupted membrane contour in green), and allow release of some of the encapsulated content. Analysis of the sulforhodamine B fluorescence intensity inside the GUV shows a 7% decrease after macropore resealing (see Figure S5D). Importantly, contrary to vesicles encapsulating agarose where residual agarose molecules block pore closure (Figures S5A and S5B, see also Ref. [2]), the membrane here fully reseals and no long-term leakage is detected, suggesting that the agarose scaffold around the vesicle does not obstruct the membrane resealing even though increasing the pore lifetime (t_{pore} ~ 160 ms). The overall vesicle deformation is stronger than that of vesicles encapsulating agarose, but weaker than that of vesicles in agarose-free medium. This outcome is understandable considering the gel-like environment of the agarose-immobilized GUVs, which does not allow for the vesicles to freely deform. The relaxation time of the immobilized vesicles is also slower than that of agarose-free vesicles, τ_{relax} ~ 1.4 s (Figure S4). This finding was reproducible for all vesicles analyzed, and observed for vesicles containing 50 or 100 mol% of POPG lipids (not shown). Apparently, the vesicle deformation induced by the pulse induces rearrangement of the agarose mesh around it, which in return slows down the relaxation of the deformed vesicle.

In summary, these results demonstrate a different behavior of GUVs subjected to mechanical strain when agarose is present in their interior or exterior. While GUV integrity is lost when agarose is present inside (Figures S5A and S5B), membrane integrity is maintained in immobilized vesicles (agarose outside, Figure S5C and S5D) and only their relaxation and pore lifetime are slowed down because of the presence of the external agarose scaffold. The results broadly validate the mechanical confinement provided by agarose and the preservation of vesicle structural integrity, allowing reliable measurements to be performed on agarose-immobilized GUVs.

S5. Diffusion of molecules to immobilized GUVs after gelation

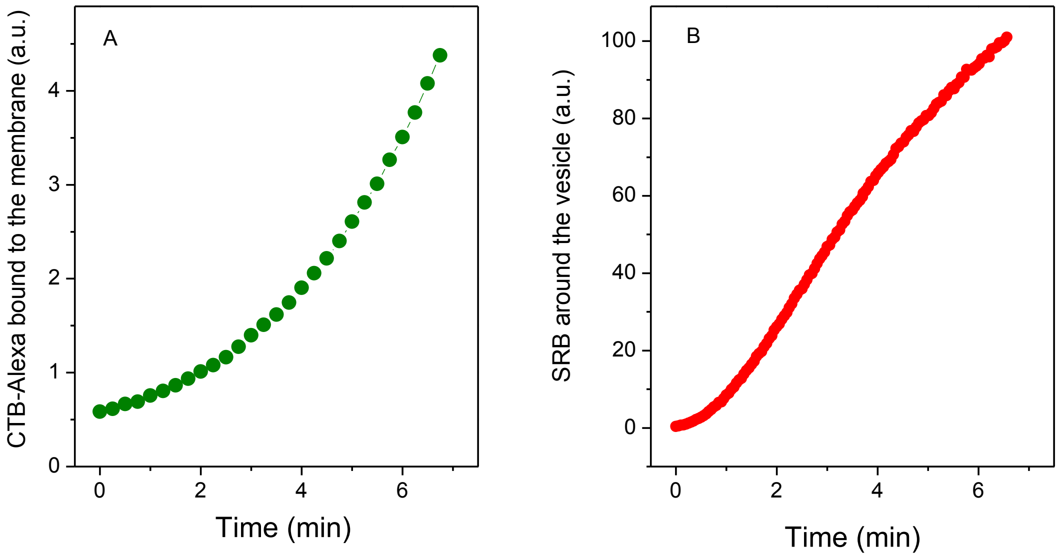

Figure S6. Time dependence of the fluorescence intensity of A) CTB-Alexa bound to the immobilized GUV and B) SRB around the GUV. The data were collected in the sequences shown in Figure 2B (A) and 2C (B) from the manuscript.

S6. Diffusion of aqueous probes in the agarose mesh measured with FCS and FRAP.

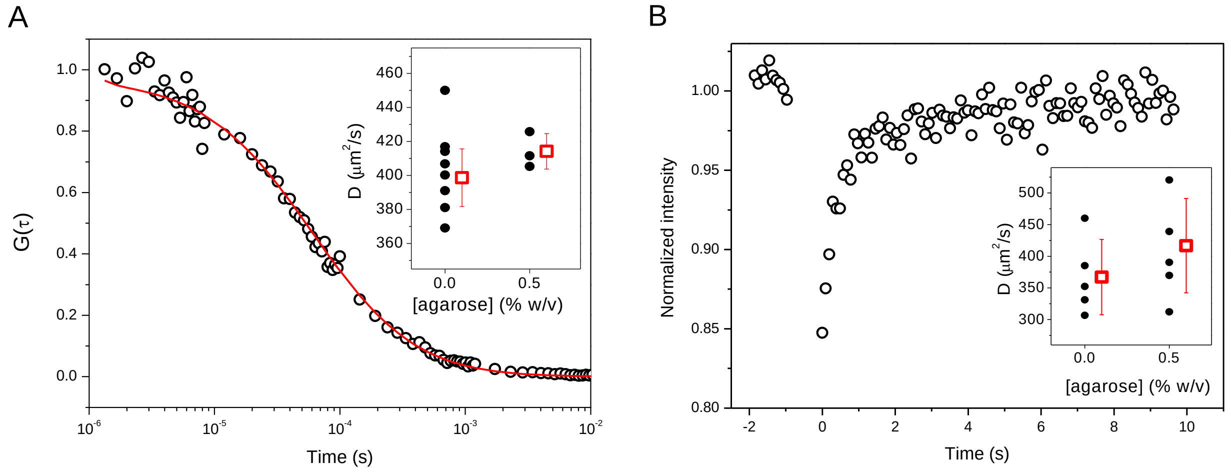

Figure S7. A) Representative normalized autocorrelation curve of 10 nM sulforhodamine B in solution (no agarose) with corresponding fit (red line). The inset shows diffusion coefficient values measured for SRB in solution with and without 0.5 % w/v agarose and the mean values with standard deviation. B) Representative FRAP recovery curve obtained for 0.2 μM carboxyfluorescein in the presence of 0.5 % w/v agarose. The photobleached area was large (20 μm diameter) to probe long-range diffusion as compared to FCS data which shows only short-range diffusion (within the confocal volume with dimensions of about 2 x 0.3 x 0.3 μm^3). Details on the FRAP analysis are given in Figure S9. The inset shows diffusion coefficients measured for CF in solution with and without 0.5 w/v agarose and the mean values with standard deviation.

Table S1. Measured diffusion coefficient D (mean value ± SD, $\mu m^2/s$) of DiI C18 (FCS) and NBD-PE (FRAP) in membranes of different lipid composition. The GUVs were immobilized in 0.5% w/v agarose.

	POPC	POPC:POPG 1:1	POPG
FCS	8.3 ± 1.8	6.4 ± 0.8	4.9 ± 0.4
FRAP	9.8 ± 1.7	8.3 ± 1.2	7.0 ± 1.0

S7. Mobility of liposomes in agarose.

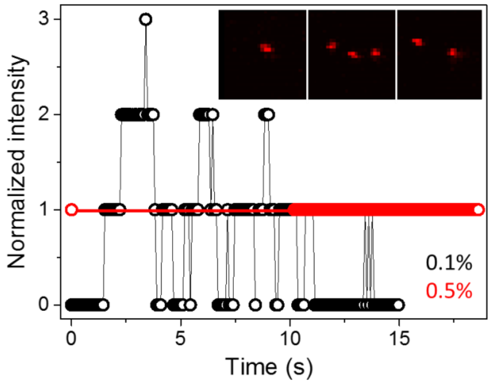

Figure S8. Quantification of liposome mobility for two agarose concentrations. The number of liposomes in a selected ROI (5 μm diameter) was counted over time and is plotted for the two agarose concentrations tested. The inset shows representative images of different number of liposomes in the ROI region imaged (5 μm x 5 μm) at 0.1% agarose. At 0.5% w/v agarose (red data), the single liposome imaged does not leave the ROI nor do other liposomes enter it.

S8. Determination of the effective radius of the bleached area

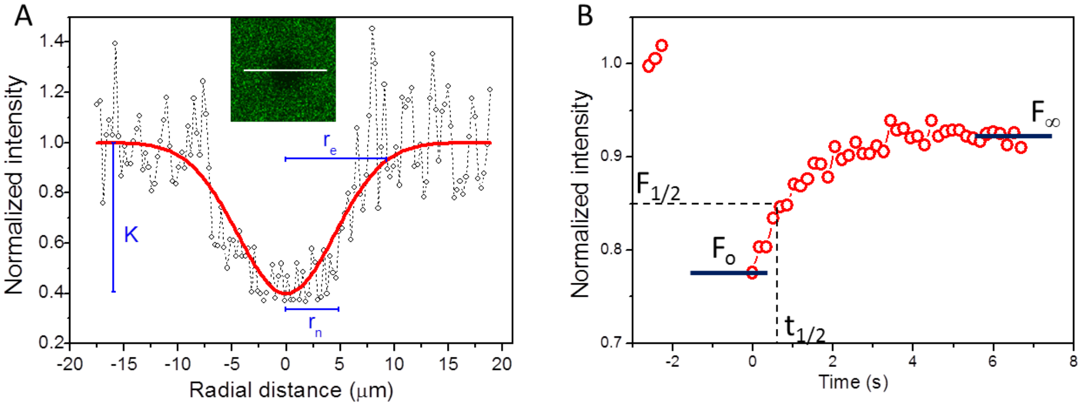

Figure S9. A) Measurement of the effective radius r_e. The intensity line profile of the first post-bleach spot is shown in black. The red line is the fit according to the equation $f(x) = 1 - K\, exp\frac{-2x^2}{r_e^2}$ (eq. 2 in the main text **and Ref.** [4]). The nominal radius (r_n = 5 μm), the effective radius (r_e = 9 μm) and the bleaching depth *K* are shown in blue. The inset shows the image used for the measurement. The effective radius was determined on bilayer patches on the cover slip surface, which resulted from GUVs that spontaneously ruptured and adhered to the glass surface in the presence of salts. This approach allowed better imaging of the bleached area. B) Typical FRAP recovery curve for a POPC GUV immobilized in 0.5% (w/v) agarose. F_o and F_∞ are the fluorescence intensities in the first post-bleach image and after recovery, respectively, and $t_{1/2}$ is the time to reach $F_{1/2} = (F_o + F_\infty)/2$.

S9. Distribution of the number of DiI C18 molecules in the FCS volume.

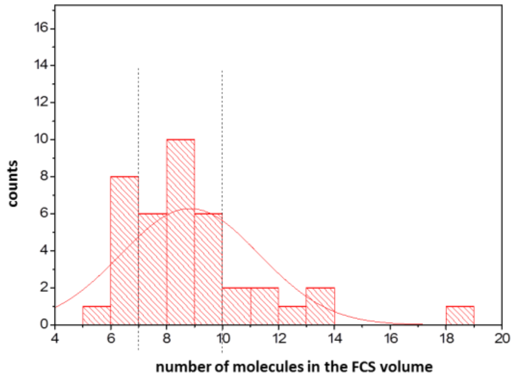

Figure S10. Histogram of the number of DiI C18 molecules in the FCS volume. The curve is a Gaussian fit of the data. Only measurements with dye concentration in the range between the dashed vertical lines were considered.

References

1 Horger, K. S., Estes, D. J., Capone, R. & Mayer, M. Films of Agarose Enable Rapid Formation of Giant Liposomes in Solutions of Physiologic Ionic Strength. *J. Am. Chem. Soc.* **131**, 1810-1819 (2009).

2 Lira, R. B., Dimova, R. & Rieke, K. A. Giant Unilamellar Vesicles Formed by Hybrid Films of Agarose and Lipids Display Altered Mechanical Properties. *Biophys. J.* **107**, 1609-1619 (2014).

3 Riske, K. A. & Dimova, R. Electro-deformation and poration of giant vesicles viewed with high temporal resolution. *Biophys. J.* **88**, 1143-1155 (2005).

4 Kang, M., Day, C. A., Kenworthy, A. K. & DiBenedetto, E. Simplified Equation to Extract Diffusion Coefficients from Confocal FRAP Data. *Traffic* **13**, 1589-1600 (2012).
